# Methyl-Coenzyme M Reductase and Its Post-translational Modifications

**DOI:** 10.3389/fmicb.2020.578356

**Published:** 2020-10-09

**Authors:** Hao Chen, Qinglei Gan, Chenguang Fan

**Affiliations:** ^1^ Cell and Molecular Biology Program, University of Arkansas, Fayetteville, AR, United States; ^2^ Department of Chemistry and Biochemistry, University of Arkansas, Fayetteville, AR, United States

**Keywords:** methyl-coenzyme M reductase, methanogenesis, post-translational modification, methylation, thioamidation, anaerobic oxidation of methane, anaerobic methanotrophic archaea, methanogenic archaea

## Abstract

The methyl-coenzyme M reductase (MCR) is a central enzyme in anaerobic microbial methane metabolism, which consists of methanogenesis and anaerobic oxidation of methane (AOM). MCR catalyzes the final step of methanogenesis and the first step of AOM to achieve the production and oxidation of methane, respectively. Besides a unique nickel tetrahydrocorphinoid (coenzyme F430), MCR also features several unusual post-translational modifications (PTMs), which are assumed to play important roles in regulating MCR functions. However, only few studies have been implemented on MCR PTMs. Therefore, to recapitulate current knowledge and prospect future studies, this review summarizes and discusses studies on MCR and its PTMs.

## Introduction

Anaerobic microbial methane metabolism is one key part of the global carbon cycle, which controls the methane emission to the atmosphere (Kirschke et al., 2013; Evans et al., 2019). Methanogenic archaea and anaerobic methanotrophic archaea (ANME) in anoxic environments are the major groups involved in microbial methane metabolism (Enzmann et al., 2018). During methanogenesis, methanogenic archaea use the end products of fermentation to produce methane anaerobically (Sieber et al., 2012; Vanwonterghem et al., 2016; Lyu et al., 2018b; Evans et al., 2019). There are three major pathways of methanogenesis, namely hydrogenotrophic, aceticlastic, and methylotrophic (Lyu and Whitman, 2019). On the other hand, ANME treat CH_4_ as a carbon source and get energy from oxidizing it into CO_2_, which is so-called anaerobic oxidation of methane (AOM; Orphan et al., 2001; Scheller et al., 2016; Timmers et al., 2017). Methanogenesis and AOM are thought to be reverse to each other based on the fact that the methyl-coenzyme M reductase (MCR) is involved in both methanogenesis and AOM, and its gene sequences are similar in both methanogenic archaea and ANME (Knittel and Boetius, 2009; Prakash et al., 2014; Ragsdale, 2014; Wang et al., 2014; Wongnate and Ragsdale, 2015). Previous studies have explored this enzyme from phylogeny to structures, reactions, and functions (Mansoorabadi et al., 2017; Thauer, 2019). As one remarkable feature of MCRs, coenzyme F430 located in the active site has been proved to be crucial, and focuses have been put on its reaction mechanism and activation (Wongnate and Ragsdale, 2015; Wongnate et al., 2016; Lyu et al., 2018a). Besides coenzyme F430, unusual post-translational modification (PTM) is identified as another remarkable feature of MCR (Kahnt et al., 2007; Mansoorabadi et al., 2017; Nayak et al., 2020). However, the biosynthesis and functions of MCR PTMs remain largely unclear. Here, we provide an introduction of MCR, and then on recent studies on PTMs of MCR.

## Methyl-Coenzyme M Reductase

MCR is the central enzyme of both methanogenesis and AOM. MCRI and MCRII are the common isozymes of MCR found in methanogenic archaea. They were firstly distinguished in *Methanothermobacter marburgensis* by anion exchange chromatography (Rospert et al., 1990). Besides *M. marburgensis*, many other species of *Methanobacteriales* and some species of other genera such as *Methanococcales* have been found to contain both MCRI and MCRII (Wagner et al., 2017). Furthermore, based on phylogenetic reconstruction of MCRs, Wagner et al. demonstrated a new type of MCR (highly structurally similar to MCRI and MCRII) exclusively from *Methanococcales*, so called MCRIII (Wagner et al., 2017). But, there is only one type of MCR found in ANME (Thauer, 2019).

### Structure Features of MCRs

MCRs contain three different subunits, α (McrA), β (McrB), and γ (McrG). And the high-resolution X-ray structures showed that MCRs usually function as a dimer of heterotrimers (α2β2γ2) with the nickel tetrahydrocorphinoid (coenzyme F430) at its active sites (Ermler et al., 1997; Wagner et al., 2017). In each heterotrimer (αβγ), three subunits are tightly associated with each other, leaving only a 50 Å long hydrophobic channel, which starts from the surface and ends with a narrow pocket containing a coenzyme F430 in the active site (Ermler et al., 1997; Thauer, 2019). Similarly, an X-ray structure of the MCR from an ANME-1 archaea also showed a dimer of a heterotrimer structure, and further analysis of this structure suggested that methanogenic and methanotrophic MCRs share the same substrates (Shima et al., 2012). However, there are also paradoxes to link methanogenic MCRs to methanotrophic MCRs, such as a coenzyme F430 modified with a methylthio group and different PTM patterns (Shima et al., 2012; Mansoorabadi et al., 2017).

### Assembly of MCRs

The MCR complex is encoded by *mcrBDCGA* operon (*mcrC* is absent in operon for MCRII), and the gene order is conserved in both methanogenic archaea and ANME (Thauer, 2019). The assembly and maturation of the MCR complex has been recently studied, and a co-translated “order assembly” model was hypothesized to explain the assembly process of MCR complex. In this model, McrB is first expressed, and forms an initial complex with McrD. The complex McrBD then associates McrG and McrA when they are translated. The McrD unit is possibly lost or weakly associated after facilitating PTMs in active site and the binding of coenzyme F430 to the modified complex (Lyu et al., 2018a). Although this model provided a possible pathway of MCR complex assembly, it was speculative, and more future studies are needed to explore the specific function of the McrD unit and its interactions with the functional part McrABG.

### Activation of MCR

The activity of MCR depends on its unique nickel-containing coenzyme F430. The nickel in coenzyme F430 can be in three different oxidation states: redox-active Ni(I), inactive Ni(II), and Ni(III) (Wongnate and Ragsdale, 2015). It was well-established that to initiate catalysis, the MCR complex must be in its active state, EPR-active {MCR_red1_ [Ni(I)-F430]}, where the nickel is in the 1+ oxidation state (Becker and Ragsdale, 1998; Thauer, 2019). *M. marburgensis* has a strong MCR_red1_ [Ni(I)-F430] signal when incubated with 100% H_2_ or CO (Rospert et al., 1991; Zhou et al., 2013). In addition, in the presence of reductant Ti(III) citrate, the MCR_ox1_ [high spin Ni(II) thiyl-radical] form (inactive) could be converted to MCR_red1_ [Ni(I)-F430] form (active; Goubeaud et al., 1997). Later, Prakash et al. performed an *in vitro* cell free activation system of MCR in the presence of dithiothreitol and protein components A2, an ATP carrier, and A3a (Prakash et al., 2014). It indicated the possible functions of protein components A2 and A3a. A2 with two ATP binding domains could serve as a carrier for ATP to Fe protein homolg and help ATP hydrolysis. A3a was a multienzyme complex containing *mcrC* gene product, and protein components involved in heterodisulfide (HDS) reductase coupled electron bifurcation (Prakash et al., 2014). However, the detailed function of each component in A3a in MCR activation still remains unclear. Furthermore, this study also demonstrated that the presence of CoM-S-S-CoB (HDS) could promote the inactivation of the enzyme (Prakash et al., 2014).

### Reaction Mechanisms of MCRs

The reaction catalyzed by MCRs involves two substrates (the methyl donor methyl-CoM and the electron donor CoB) and two products (CH_4_ and CoM-S-S-CoB). In the methanogenesis process, the final reaction occurs when substrates bind to the substrate channel at the active site, where methyl-CoM is proximal to the nickel in coenzyme F430 (Wongnate et al., 2016). However, the exact mechanism to explain the reaction at the active site has not been fully solved. There are three main proposed mechanisms: mechanism I involves a generation of a methyl-Ni(III) intermediate (Horng et al., 2001); mechanism II assumes the production of a Ni(II)-thiolate and a methyl radical (Scheller et al., 2013; Wongnate et al., 2016); and mechanism III indicates the existence of intermediates, a highly reactive methyl anion and Ni(III)-SCoM (Harmer et al., 2005). A recent study conducted by Wongnate and Ragsdale elucidated the order of reactions at the active site of MCR based on the kinetic approach (Wongnate and Ragsdale, 2015). The binding of methyl-CoM to nickel in coenzyme F430 triggers the movement of CoB, then generates the enzyme-substrate complex [CoB·MCR(NiI)·CH_3_-CoM], and releases CH_4_. Later, Wongnate and Ragsdale applied transient kinetic, spectroscopic, and computational approaches and obtained results more consistent with mechanism II, the methyl radical mechanism (Wongnate et al., 2016). On the other direction, although studies also suggested that the first step of AOM could also be the rate-limiting step (Scheller et al., 2010), the reaction mechanism of MCR in ANME was rarely studied, either from the kinetic or structural aspect.

## Post-Translational Modifications of MCRs

**Figure 1:**
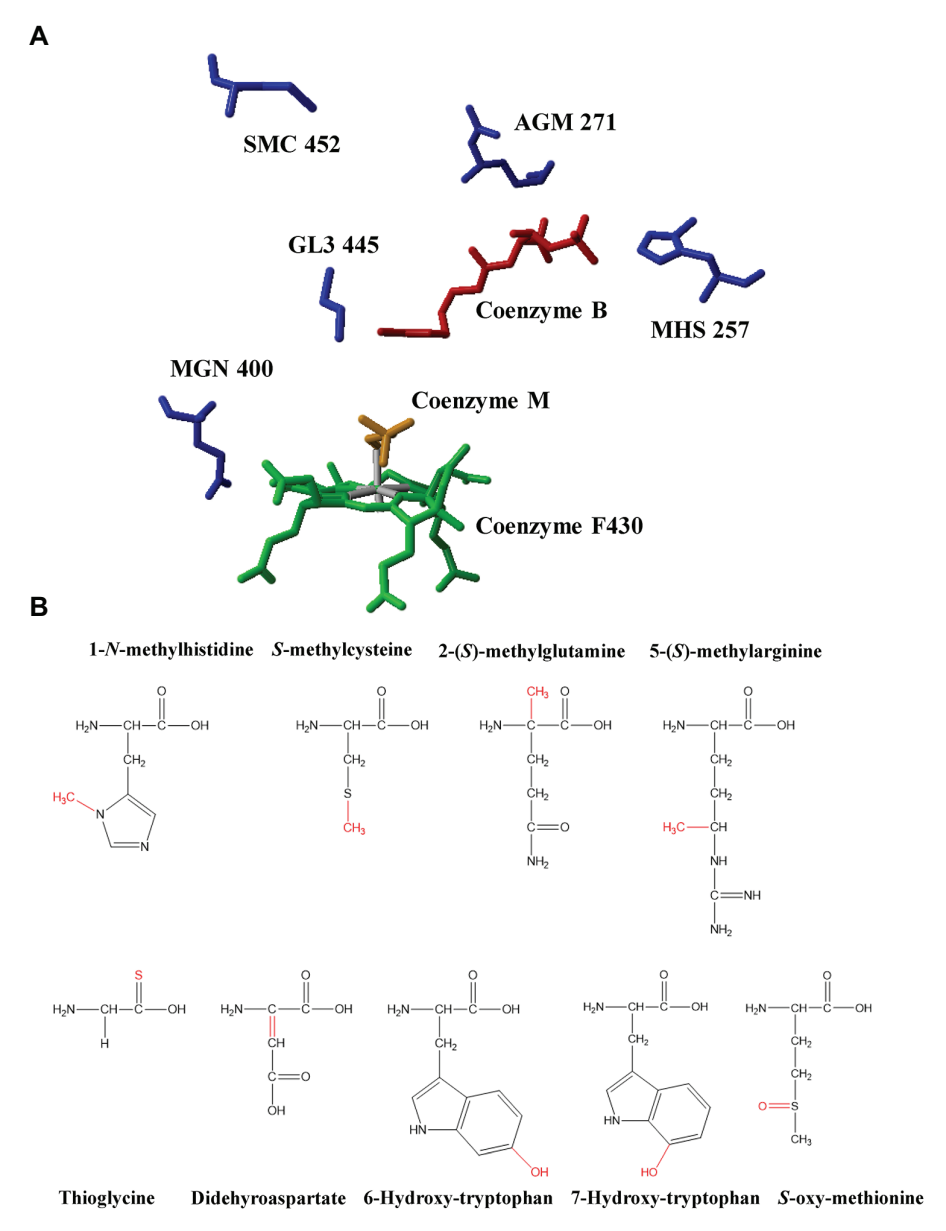
**(A)** The active site of methyl-coenzyme M reductase (MCR) from *Methanothermobacter marburgensis* and identified post-translational modifications (PTMs; PDB ID: 1MRO, Ermler et al., 1997). Red: coenzyme B; Yellow: coenzyme M; Green: F430; AGM: 5-(S)-methyl-Arg^271^; MHS: 1-N-methyl-His^257^; GL3: thio-Gly^445^; MGN: 2-(S)-methyl-Gln^400^; and SMC: S-methyl-Cys^452^. **(B)** The chemical structures of PTMs identified in methyl-coenzyme M reductases from methanogenic archaea and anaerobic methanotrophic (ANME) archaea. Modifications were marked with red color.

Unusual PTMs are another remarkable feature of MCRs. The first observation of PTMs in MCR dated back to 1997 when Ermler et al. determined the first high-resolution crystal structure of MCRI in a *Methanobacterium thermoautotrophicum*. Their study demonstrated the existence of five PTMs at the active sites of MCR (Ermler et al., 1997). So far, 1-*N*-methylhistidine, *S*-methylcysteine, 2-(*S*)-methylglutamine, 5-(*S*)-methylarginine, thioglycine, didehydroaspartate, and 6-hydroxy-tryptophan have been found in methanogenic MCRs (Ermler et al., 1997; Selmer et al., 2000; Wagner et al., 2016, 2017; Mansoorabadi et al., 2017). Among these PTMs, arginine methylation can be found in all methanogenic archaea under laboratory conditions but not ANME like ANME-1 (Kahnt et al., 2007). Cysteine methylation is in a low abundance or absent in many methanogenic archaea, such as *M. maripaludis*. Glutamine methylation is absent in *Methanosarcina barkeri* (Selmer et al., 2000; Lyu et al., 2018a). Didehyroaspartate is absent in *Methanothermobacter wolferi* (Wagner et al., 2016). Although methanotrophic MCRs share two conserved PTMs, 1-*N*-methylhistidine and thioglycine with methanogenic archaea, they harbor their own PTMs, 7-hydroxy-tryptophan and *S*-oxymethionine (Shima et al., 2012; Wagner et al., 2017; Figure 1; Table 1).

**Table 1:** Recent findings on the biosynthesis and functions of PTMs in the active site of MCRs.

PTMs	Biosynthesis	Functions
Methanogens		
1-*N*-methylhistidine	SAM-dependent N-methyltransferase (No exact enzymes found yet)	Might serve to position the imidazole that coordinates CoB (Grabarse et al., 2000)
*S*-methylcysteine	SAM-dependent S-methyltransferase (*mcmA* gene; Nayak et al., 2020)	Might play a role in adaption to mesophilic conditions (Nayak et al., 2020)
2-(*S*)-methylglutamine	Putative cobalamin-dependent radical SAM enzyme (No evidence yet)	Further studies needed
5-(*S*)-methylarginine	Mmp10 protein (*mmpX* gene; Deobald et al., 2018; Radle et al., 2019; Lyu et al., 2020)	Play multiple roles in the catalysis, assembly or stability of MCR (Lyu et al., 2020)
Thioglycine	YcaO and TfuA (*ycaO* and *tfuA* gene; Nayak et al., 2017; Mahanta et al., 2018)	Different opinions exist regarding to catalysis or stability of MCR (Grabarse et al., 2001; Horng et al., 2001; Nayak et al., 2017)
Didehydroaspartate	Further studies needed	Found in MCRI and II, and slightly increased the catalytic efficiency of the enzyme (Wagner et al., 2016)
6-hydroxy-tryptophan	Further studies needed	Found in MCRIII, and might have equivalent role to the didehydroaspartate in MCRI and II (Wagner et al., 2017)
ANME		
1-*N*-methylhistidine	SAM-dependent *N*-methyltransferase (No exact enzyme found yet)	Might serve to position the imidazole that coordinate CoB (Grabarse et al., 2000)
Thioglycine	YcaO and TfuA (*ycaO* and *tfuA* gene; Nayak et al., 2017; Mahanta et al., 2018)	Different opinions exist regarding to catalysis or stability of MCR (Grabarse et al., 2001; Horng et al., 2001; Nayak et al., 2017)
7-hydroxy-tryptophan	Further studies needed	Assumed to compensate the absence of arginine methylation in ANMEs (Shima et al., 2012)
*S*-oxymethionine	Further studies needed	Further studies needed

### Methylation

Widely occurring at the active site of methanogenic MCRs, methylation has been identified at four different amino acid side chains, including histidine, arginine, glutamine, and cysteine. These four types of methylation are also the first group of PTMs found in MCRs (Ermler et al., 1997). Several studies have been implemented to study their biosynthesis. Firstly, Selmer et al. ruled out the possibility that the methyl groups are donated from methyl-CoM during the reaction at the active site of MCRs, and they confirmed that *S*-adenosylmethionine (SAM) was the most likely methyl donors. This was based on the observation that after culturing *M. thermoautotrophicum* in the media with l-(methyl-D_3_) methionine, the D_3_-labeled pattern was the same as two methyl groups known to be introduced by SAM in coenzyme F430 while the SAM was not used as the carbon source of methanogenesis and converted into methane (Selmer et al., 2000). This study also indicated that the biosynthesis of 1-*N*-methylhistidine and *S*-methylcysteine was chemically possible to process *via* the methyl group transfer from SAM to substitute the hydrogen of a thiol group or a ring nitrogen (nucleophilic substrates). However, this mechanism could not explain the occurrence of 2-(*S*)-methylglutamine and 5-(*S*)-methylarginine due to the low nucleophilicity of the target carbon (Selmer et al., 2000). Moreover, it was indicated that the biosynthesis of 1-*N*-methylhistidine and *S*-methylcysteine was probably catalyzed by SAM-dependent *N*- and *S*-methyltransferases by which the methyl group is transferred to *N*- or *S*-nucleophiles of histidine or cysteine *via* a S_N_2 reaction. The gene (*mcmA*) encoding the SAM-dependent methyltransferase for cysteine methylation has been recently identified, and it has been indicated that cysteine methylation might play a role in adaption to mesophilic conditions (Nayak et al., 2020). However, 2-(*S*)-methylglutamine and 5-(*S*)-methylarginine could not be formed by this mechanism (Liscombe et al., 2012; Nayak et al., 2020).

To identify the mechanism for glutamine and arginine methylation, radical SAM methyltransferases were assumed, since they have the ability to transfer methyl groups to the electrophilic sp2- or sp3-carbon centers of substrates (Bauerle et al., 2015). Based on this hypothesis, methanogenesis marker protein 10 (Mmp10), a candidate of methyltransferase for 5-(*S*)-methylarginine was selected as the target to test. *Methanosarcina acetivorans* with the deletion of ma4551 (the gene for Mmp10) showed a loss of 5-(S)-methyl-arginine^285^ at the active site of MCR, and further investigation indicated that the lack of methylation in arginine^285^ could influence the thermal stability of MCR and also cause the growth defects under stress conditions, such as oxidative stress and elevated temperature stress (Deobald et al., 2018). Additionally, an *in vitro* assay of Mmp10 from *M. acetivorans* suggested that besides SAM, cobalamin was also a cofactor required for Mmp10 activity (Radle et al., 2019). Very recently, by knocking out *mmpX* gene (encoding Mmp10 in *M. maripaludis*), Lyu et al. demonstrated a direct link between Mmp10 and arginine methylation based on the fact that the methylation of Arg^275^ was absent at the active site of MCR in mutant *M. maripaludis*. This study was the first to link MCR activity and a specific PTM. It also firstly demonstrates that a PTM could affect both methanogenesis and cell growth without the addition of stressor (Lyu et al., 2020). Furthermore, this study also indicated a high specificity of Mmp10 to McrA and the binding sites of cofactors, SAM, and cobalamin, in Mmp10 (Lyu et al., 2020). For glutamine methylation, although the putative cobalamin-dependent radical SAM enzyme has been proposed (Deobald et al., 2018), there has been no solid evidence. The functions of methylation in MCRs are not fully addressed with only few studies. Histidine methylation is theoretically thought to serve to position the imidazole ring that coordinates CoB (Grabarse et al., 2000), while arginine methylation at the active site of MCR could play multiple roles in the catalysis, assembly, or stability of the enzyme (Deobald et al., 2018; Lyu et al., 2020).

### Thioamidation

Thioamides are very rare in nature, among which thioamidation of glycine in MCRs is the only example identified in proteins (Mahanta et al., 2018). Thioamidation commonly occurs in both methanogenic and methanotrophic MCRs. The biosynthesis of thioglycine has been hypothesized to involve two proteins, YcaO and TfuA. One recent study combined genetic approaches and mass spectrometry analyses to test the possible roles of YcaO and TfuA proteins in glycine thioamidation, proving that the removal of these two genes could cause the absence of thioamidation of glycine^465^ at the active site of MCR (Nayak et al., 2017). Later, Mahanta et al. used *in vitro* reconstitutions of thioamidation, using TfuA from *M. acetivorans* and YcaO from *M. acetivorans*, *Methanopyrus kandleri*, or *Methanocaldococcus jannaschii*, to demonstrate that thioamidation of glycine could proceed *via* backbone *O*-phosphorylation. This reaction is ATP-dependent and requires an external sulfide source (Mahanta et al., 2018). As for the function of thioamidation in MCRs, there are different opinions: (1) it facilitates the catalysis as an intermediate electron carrier during the reaction (Horng et al., 2001); (2) it reduces the pKa of sulfhydryl group to assist the deprotonation of CoB (Grabarse et al., 2001); and (3) it enhances the enzyme stability rather than facilitating catalysis (Nayak et al., 2017). Recently, to test the interactions between arginine methylation, cysteine methylation and thioamidation, Nayak et al. established knock-out strains with all possible combination, and observed that the *ΔycaO-tfuA*/*ΔmcmA* double mutant strain had most severe growth, while the one with triple deletion showed a faster growth, suggesting that unmodified Arg^285^ could alleviate changes brought by the absence of thioglycine and methyl-cysteine. In addition, by phenotypic analyses, they showed that interactions between PTMs could cause unpredictable changes of the thermal stability of MCR, which might influence the activity and efficiency of MCRs (Nayak et al., 2020).

### Other PTMs

Other PTMs in MCRs are rarely studied. Didehydroaspartate, the most recent PTM found in methanogenic MCRI and MCRII was identified to be adjacent to thioglycine based on mass spectrometry analyses and high-resolution X-ray crystallography (Wagner et al., 2016). This PTM was thought to be dispensable due to the fact that it was present in MCRs of *M. marburgensis* and *M. barkeri*, but not in MCR of *Methanothermobacter wolfeii*, which is phylogenetically closer to *M. marburgensis*. However, it was also shown that didehydroaspartate could slightly increase the catalytic efficiency of the enzyme (Wagner et al., 2016). Unlike didehydroaspartate, hydroxytryptophan was found in both methanogenic archaea and ANME. 7-hydroxy-tryptophan at the active site of MCR from ANME-1 was assumed to compensate the absence of arginine methylation (Shima et al., 2012), while 6-hydroxy-tryptophan at the active site of MCRIII from *Methanotorris formicicus* was thought to have a role equivalent to the didehydroaspartate in MCRI and MCRII (Wagner et al., 2017). Till now, there are no proposed mechanisms for the biosynthesis of these PTMs yet, and no enzyme has been characterized to be related to them.

## Discussion

Recent studies have changed our view of the MCR from its evolutionary perspective to its two remarkable features, the conenzyme F430 and unusual PTMs at the active site. Recently, a study has provided supportive evidence for the methyl radical mechanism (Wongnate et al., 2016), which was only based on methanogenesis, so more future studies are necessary to explore the reaction mechanism for coenzyme 430 during the AOM. As for unusual PTMs, studies have focused on its biosynthesis and function. Histidine and cysteine are possibly catalyzed by SAM-dependent N- and S-methyltransferases, respectively, while arginine and glutamine seem to have different enzymatic methylation (Selmer et al., 2000). The methylation of cysteine and arginine have been shown to be related to a dedicated SAM-dependent (encoding by *mcmA*) and a potential radical SAM-dependent methyltransferase (Mmp10, encoding by *mmpX*); however, the enzyme for histidine and glutamine modification still remains unknown (Deobald et al., 2018; Nayak et al., 2020). YcaO and TfuA were found to be involved in the biosynthesis of thioglycine. An elucidation of the mechanisms or enzymes for other PTMs like didehydroaspartate and hydroxytryptophan at the active site of MCR still requires further studies. Moreover, although the functions of PTMs are indicated to be related to the catalysis and stability of MCR, no consistency has been given on the exact functions of each PTM. Furthermore, the results of functional studies of PTMs may vary in different species, which have different growth features.

## Author Contributions

HC, QG, and CF wrote and edited the manuscript. All authors contributed to the article and approved the submitted version.

### Conflict of Interest

The authors declare that the research was conducted in the absence of any commercial or financial relationships that could be construed as a potential conflict of interest.
